# Acetylsalicylic acid disrupts SARS-CoV-2 spike protein glycosylation and selectively impairs binding to ACE2

**DOI:** 10.3389/fimmu.2025.1706997

**Published:** 2026-01-07

**Authors:** Luca Perico, Alessandra Bovio, Susanna Tomasoni, Piera Trionfini, Domenico Cerullo, Daniela Corna, Anna Pezzotta, Monica Locatelli, Marta Alberti, Ariela Benigni, Giuseppe Remuzzi

**Affiliations:** Department of Molecular Medicine, Istituto di Ricerche Farmacologiche Mario Negri IRCCS, Bergamo, Italy

**Keywords:** COVID-19, SARS-CoV-2 spike protein, ACE2 binding, acetylsalicylic acid, glycosylation

## Abstract

Preclinical and clinical evidence suggested the potential benefits of treatment with acetylsalicylic acid (ASA) in mitigating COVID-19 severity. While available studies largely focused on the intracellular pathways through which ASA impairs viral replication or dampens host immunoresponse stimulated by SARS-CoV-2, whether ASA directly affects the interaction between the viral spike protein and its cellular receptor angiotensin converting enzyme 2 (ACE2) remains unexplored. This question is clinically relevant, as circulating spike S1 has been shown to persist in patients with acute and long COVID-19, where its interaction with the broadly expressed ACE2 drives systemic manifestations and tissue damage. Here, we demonstrate that pre-incubation of the SARS-CoV-2 spike subunit 1 (S1) with ASA dose-dependently impaired ACE2 binding on Vero cells. The functional relevance of this finding was confirmed in transgenic mice with human ACE2, in which intratracheal administration of ASA-treated S1 markedly reduced lung injury, fibrosis, and inflammation compared to untreated S1. Glycoproteomic profiling revealed that ASA altered the glycosylation landscape of S1, particularly N-glycosylation at N61 and O-glycosylation at S325. Site-directed mutagenesis of these two residues confirmed the critical role of their glycosylation in S1-ACE2 binding *in vitro*. Consistently, the glycosylation-insensitive S1 had limited effect in inducing lung injury, fibrosis, and inflammation in transgenic mice compared to WT S1, phenocopying the protective effects of ASA. These findings unveil a previously unrecognized antiviral activity of ASA, providing a molecular rationale for its repurposing as a low-cost, readily available intervention to prevent the progression from mild to severe COVID-19.

## Introduction

Coronavirus disease 2019 (COVID-19) is a communicable disease caused by SARS-CoV-2. In approximately 80-90% of infected individuals, SARS-CoV-2 infection is mainly restricted to the upper airways and manifests as a self-limiting illness with mild flu-like symptoms (1). In the remaining cases, an altered immune response facilitates uncontrolled viral replication in the lower airways, resulting in pneumonia and acute respiratory distress syndrome (2, 3). In these severe forms, systemic dissemination of the virus to distal organs contributes to endothelial injury, microthrombotic complications (4–8), multiorgan failure (9–11), and death.

Intervening at the onset of mild to severe COVID-19 in the outpatient setting provides the opportunity to prevent disease progression and long-term complications (12). Recent clinical evidence indicates that anti-inflammatory drugs, particularly non-steroidal anti-inflammatory drugs (NSAIDs), represent a promising therapeutic approach to limit COVID-19 severity (13). Indeed, several observational studies have evaluated NSAIDs, especially selective cyclooxygenase-2 (COX-2) inhibitors, as part of multipharmacological protocols for early outpatient treatment (14, 15).

Among these compounds, aspirin (acetylsalicylic acid, ASA) has been shown to reduce in-hospital mortality and severe outcomes in COVID-19 patients, as reported in several retrospective analyses and meta-analyses involving thousands of patients (16–31), although with mixed results (32, 33). The primary mechanism of action of ASA is attributed to its ability to acetylate serine residues in the active site of COX-2 and COX-1, thereby inhibiting their activity (34). By targeting COX-2, ASA reduces the production of prostaglandins, which are critical mediators of the inflammatory response. Simultaneously, inhibition of COX-1 in platelets diminishes thromboxane A2 synthesis (34), a key molecule involved in promoting platelet aggregation and vasoconstriction. This antithrombotic effect has positioned ASA as a candidate for preventing thromboembolic events in severe disease (35), which are strongly associated with COVID-19 mortality risk (4–8).

Beyond these anti-inflammatory and anti-thrombotic effects, ASA has demonstrated direct antiviral activity against various RNA viruses, including hepatitis (36–38), flavivirus (39), rotavirus (40), and respiratory viruses (41–43), as well as DNA viruses such as varicella zoster (44, 45) and cytomegalovirus (46). In the context of COVID-19, evidence indicates that ASA inhibits SARS-CoV-2 replication both in cell culture and in *ex vivo* models using human precision-cut lung slices (47). These studies revealed that ASA exerts antiviral activity through multiple mechanisms, including inhibition of viral replication, acetylation of viral or host proteins, suppression of NF-κB signaling, and modulation of host metabolic and post-translational pathways, ultimately limiting viral transcription and assembly.

While these findings primarily focused on the intracellular mechanisms by which ASA modulates viral replication or attenuates host cell responses, it remains unclear whether ASA can directly interfere with the interaction between the viral spike protein and its receptor angiotensin converting enzyme 2 (ACE2). Blocking the S1–ACE2 interaction is particularly important, as high levels of circulating SARS-CoV-2 antigens, particularly the spike protein S1 subunit (S1), have been detected in patients with severe disease (48, 49). In addition, the widespread distribution of ACE2 across multiple organs enables receptor engagement by S1 and activation of detrimental signaling pathways, thereby contributing to systemic symptoms and multi-organ involvement, such as cardiopulmonary manifestations (48, 49). Additionally, circulating S1 has been detected in the post-acute sequelae of long-COVID (50, 51). As an example, persistence of spike protein at the skull-meninges-brain axis has been associated with the neurological sequelae of long-COVID (52). Therefore, blocking spike–ACE2 interaction during SARS-CoV-2 infection may have therapeutic relevance in mitigating systemic complications beyond viral entry in the respiratory tract.

In this study, we sought to investigate the potential effects of ASA on the spike protein of SARS-CoV-2, specifically assessing its ability to interfere with spike–ACE2 binding. Clarifying this potential mode of action is critical in the context of repurposing ASA as an affordable therapeutic option, as disrupting spike–ACE2 binding could prevent the downstream cascade leading to organ injury, thrombosis, and long-term complications associated SARS-CoV-2 infection (53–55).

## Results

### Treatment with ASA reduces cell surface binding of recombinant SARS-CoV-2 S1 protein in a dose-dependent manner

In an initial attempt to assess the ability of ASA to alter S1 binding to ACE2, we developed an *in vitro* system using Vero cells exposed to recombinant S1 subunit (S1) of the spike protein of SARS-CoV-2, which contains the receptor binding domain (RBD) for ACE2 interaction (56, 57). First, we assessed the impact of different concentrations of the S1 protein on Vero cells. In this setting, an irrelevant isotype antibody or a functional blocking antibody targeting ACE2 were used to determine whether this toxicity was dependent on S1-ACE2 binding. As shown in **Supplementary Figure 1A**, the exposure of Vero cells to increasing concentrations of S1 (10, 20, and 50 nM) in the presence of an irrelevant isotype antibody induced a dose-dependent decrease in cell viability after 24 hours. In contrast, co-incubation with the anti-ACE2 blocking antibody effectively prevented cell toxicity at 10 and 20 nM (**Supplementary Figure 1A**). However, 50 nM S1 still induced a significant reduction in cell viability despite ACE2 blockade (**Supplementary Figure 1A**), suggesting that, at this dose, the S1 cytotoxicity may occur *via* ACE2-independent mechanisms, such as off-target interactions or non-specific cytotoxic effects potentially arising from supraphysiological ligand levels.

**Figure 1:**
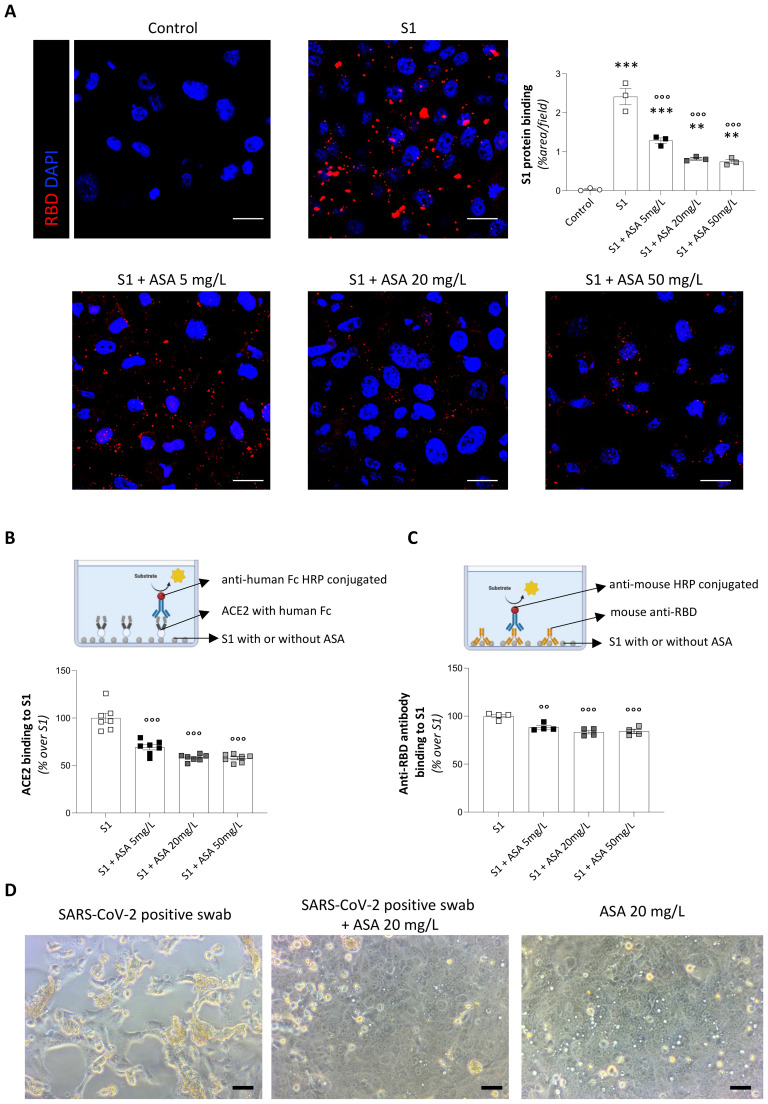
ASA inhibits S1-ACE2 binding in Vero cells. **(A)** Representative images and quantification of the binding of spike protein (RBD, red) on Vero cells following 24 h treatment with 20 nM S1 incubated overnight with control medium (S1) or increasing concentrations of ASA in control medium (S1+ASA 5 mg/L, S1+ASA 20 mg/L, and S1+ASA 50 mg/L). Cell treated with medium alone served as control. Nuclei were counterstained with DAPI (blue). Data are expressed as % of positive area *per* high power field at ×63 magnification (% area/field, n=3 *per* group). Scale bar 20 µm. **(B)** Schematic representation (upper panel, created with BioRender.com) and quantification (lower panel) of the ELISA assay used to evaluate the binding of ACE2 to S1 protein treated overnight with PBS 1X (S1) or with increasing concentrations of ASA in PBS 1X (S1+ASA 5 mg/L, S1+ASA 20 mg/L, and S1+ASA 50 mg/L). Data are expressed as percentage of relative binding compared to S1 (n=7 *per* group). **(C)** Schematic representation (upper panel, created with BioRender.com) and quantification (lower panel) of the ELISA assay used to evaluate the binding of an anti-S1 antibody to S1 protein treated overnight in PBS 1X (S1) or with increasing concentrations of ASA (S1+ASA 5 mg/L, S1+ASA 20 mg/L, and S1+ASA 50 mg/L). Data are expressed as percentage of relative binding compared to S1 (n=4 *per* group). **(D)** Representative phase-contrast images showing the cytopathic effects in Vero cells at 4 days following exposure to SARS-CoV-2-positive swab incubated for 1 h with control medium (SARS-CoV-2 positive swab) or with ASA 20 mg/L (SARS-CoV-2 positive swab + ASA 20 mg/L). Cell treated with ASA 20 mg/L served as control. Scale bar 50 µm. Results are shown as mean ± SEM and were analyzed with Tukey’s multiple comparison test. **p-value<0.01, and ***p-value<0.001 *vs* control; °°p-value<0.01, and °°°p-value<0.001 *vs* S1.

Having identified the S1 concentrations that induce ACE2-dependent toxicity, we selected to use 20 nM S1 for subsequent experiments. Using immunofluorescence analysis, we observed a significant binding of the 20 nM S1 protein to the apical surface of Vero cells (**Supplementary Figure 1B**). In this setting, we investigated whether ASA could reduce the binding affinity of S1. To this aim, S1 protein was incubated overnight with increasing concentrations of ASA. To ensure clinical relevance, we used three different concentrations of ASA (5, 20, and 50 mg/L), in the range of plasma levels achievable with oral doses of 500–1000 mg ASA (58). As shown in **Figure 1A**, pre-treatment with ASA was able to significantly inhibit the binding of S1 to Vero cells in a dose-dependent manner. Of note, this inhibitory effect was not observed when S1 was incubated with another compound, namely paracetamol (**Supplementary Figure 2**), indicating the specific effect of ASA.

To further confirm the direct role of ASA in blocking the interaction between S1 and ACE2, we established an ELISA-based binding assay, adapted with minor modifications from previously published protocols (59). Specifically, S1 protein was treated or not with increasing concentrations of ASA (5, 20, and 50 mg/L) and then immobilized on ELISA plates (**Figure 1B**, upper panel). Following incubation with recombinant human ACE2 fused to a human Fc domain, binding of ACE2 to S1 was detected using an HRP-conjugated anti-human Fc secondary antibody (**Figure 1B**, upper panel). In this setting, ASA treatment was sufficient to impair S1-ACE2 binding efficiency in a dose-dependent manner, with a reduction of up to 50% at a dosage of 20 mg/L (**Figure 1B**, lower panel). This experimental setup also enabled us to assess the direct effect of ASA on the inhibition of ACE2–S1 interaction, while eliminating any potential confounding influence of ASA on the binding between S1 and the detection antibody. This latter effect was indeed marginal, as aspirin only partially limited the recognition of S1 by a specific anti-RBD antibody and this effect was independent of its dosage (**Figure 1C**).

Overall, these results demonstrate that ASA specifically impairs the binding of the S1 protein to ACE2 in a dose-dependent manner, both in cell-based and cell-free systems, supporting a direct effect of ASA on the molecular interaction between S1 and ACE2.

### Treatment with ASA inhibits binding of replication-competent SARS-CoV-2, limiting its cytopathic effect

To further validate the potential direct impact of ASA in disrupting the interaction between the spike protein and ACE2, we performed a cytopathic assay in Vero cells with replication-competent SARS-CoV-2. As depicted in the phase-contrast micrographs (**Figure 1D**, left panel), inoculation with the SARS-CoV-2 positive nasopharyngeal swab sample alone induced a distinct and extensive cytopathic effect in Vero cells after 4 days, characterized by widespread cellular rounding, syncytia formation, and detachment from the culture substrate, indicative of significant viral replication and subsequent cellular damage. Large areas of the cell monolayer exhibited marked disruption, consistent with the expected pathological effects of SARS-CoV-2 infection in susceptible cells. In contrast, when the SARS-CoV-2 positive viral sample was pre-incubated with 20 mg/L ASA, the cytopathic effect was strikingly abrogated (**Figure 1D**, middle panel). Under these conditions, the Vero cell monolayer largely maintained its integrity, exhibiting a healthy, confluent appearance with characteristic flat, polygonal cell morphology, comparable to that of uninfected control cells. Importantly, ASA alone had no detectable impact on the integrity of the Vero cell monolayer (**Figure 1D**, right panel).

Collectively, these results demonstrate the ability of ASA to attenuate the cytopathic effects induced by SARS-CoV-2 in Vero cells, highlighting its potential to block virus–host cell interactions in susceptible cells.

### Treatment with ASA limits the pathogenic effect of S1 in transgenic mice with human ACE2

**Figure 2:**
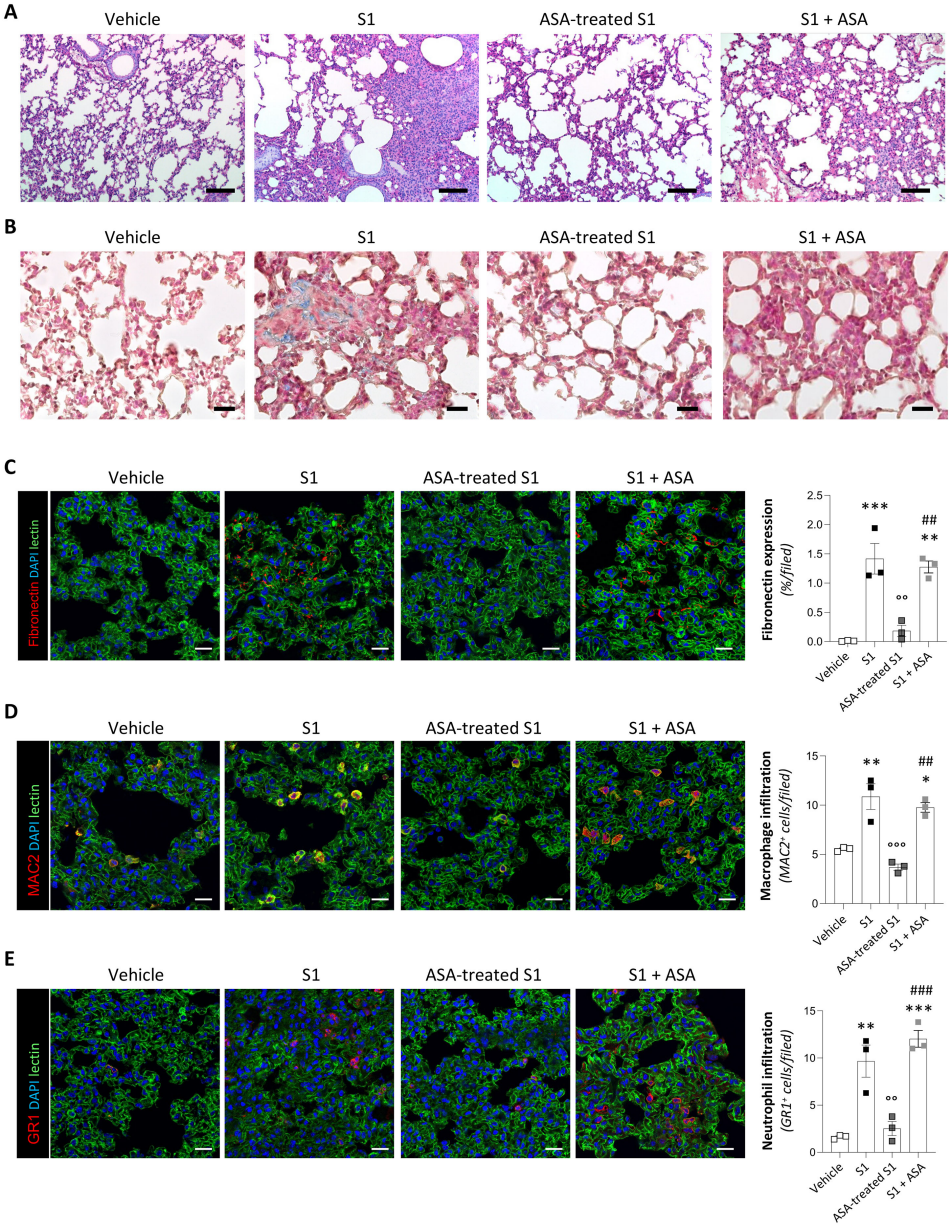
ASA prevents S1-induced lung injury, fibrosis and inflammation in hACE2-KI mice. **(A, B)** Representative images of lung sections stained with H&E **(A)** and Masson’s trichrome **(B)** from mice receiving intratracheal instillation of vehicle, 15 μg S1 pre-treated overnight with vehicle (S1), 15 μg S1 pre-treated overnight with ASA 20 mg/L (ASA-treated S1) or 15 μg S1 pre-treated overnight with vehicle followed by ASA 20 mg/L administered immediately afterward through the same route (S1+ASA) at 7 days (n=3 *per* group). Scale bars: 100 µm for H&E and 20 µm for Masson’s trichrome staining. **(C-E)** Representative images and quantification of fibronectin (**C**, red), MAC2^+^ macrophages (**D**, red), and GR1^+^ neutrophils (**E**, red) in lung tissue sections of mice receiving intratracheal instillation of vehicle, S1, ASA-treated S1 or S1+ASA at 7 days (n=3 *per* group). Lung structures and nuclei were counterstained with WGA lectin (green) and DAPI (blue), respectively. Scale bar: 20 µm. Data are expressed as % of fibronectin fluorescence area *per* high power field at ×63 magnification (% area/field) and the average number of MAC2^+^ or GR1^+^ cells *per* high power field at ×63 magnification. For all panels, results are shown as mean ± SEM and were analyzed with Tukey’s multiple comparison test. *p-value<0.05, **p-value<0.01, and ***p-value<0.001 *vs* Vehicle; °°p-value<0.01, and °°°p-value<0.001 *vs* S1; ^##^p-value<0.01, and ^###^p-value<0.001 *vs* ASA-treated S1.

To validate whether treatment of S1 with ASA blocks its binding to ACE2, thereby limiting organ damage, we employed a biologically relevant *in vivo* model of acute lung injury induced by SARS-CoV-2 S1 protein in transgenic mice expressing human ACE2 (hACE2-KI). To this end, mice received an intratracheal instillation of 15 µg S1 pre-incubated overnight in saline or 20 mg/L ASA. Hematoxylin and eosin staining of lung sections from mice instilled with S1 incubated with saline revealed significant histological changes at 7 days compared to hACE2-KI mice receiving vehicle (**Figure 2A**). In particular, S1-treated mice showed dilation of the alveolar spaces and increased thickness of the alveolar septa, associated with lung tissue hypercellularity within the alveolar parenchyma and in blood vessels, perivascular sites and spreading to larger interstitial areas (**Figure 2A**). These histological abnormalities were significantly attenuated in the lungs of hACE2-KI mice receiving S1 pre-incubated with ASA (**Figure 2A**).

To further evaluate the impact of ASA on the S1 pathogenicity in the lungs, Masson’s Trichrome staining was performed to assess extracellular matrix remodeling across the experimental groups. Lungs from mice receiving vehicle exhibited well-preserved alveolar structures with no matrix deposition (**Figure 2B**). Conversely, mice instilled with S1 pre-incubated with saline displayed substantial and diffuse fuchsin-positive signal, reflecting intense extracellular matrix accumulation, as well as aniline blue staining, indicating increase in mature collagen fibers (**Figure 2B**). Pre-incubation of S1 with ASA reduced both fuchsin- and aniline-positive areas in the lungs of hACE2-KI mice (**Figure 2B**), suggesting the ability of ASA to reduce S1-induced pro-fibrotic processes (60).

To gain deeper insights into how ASA treatment affects the capacity of S1 to induce fibrosis, we examined the expression of fibronectin, a hallmark of nascent fibrotic remodeling that serves as the dominant matrix component providing a scaffold for collagen deposition (61). Immunofluorescence analyses revealed a significant increase in fibronectin expression in the lung of mice treated with S1 pre-incubated with saline compared to mice receiving vehicle (**Figure 2C**). Notably, S1 pre-incubated with ASA exhibited reduced pro-fibrotic activity, as evidenced by a significantly reduced expression of fibronectin in the lungs of hACE2-KI mice (**Figure 2C**).

We next investigated whether these effects functionally translated into a reduction in inflammatory cell infiltrates. To achieve this, we performed immunofluorescence analysis of MAC2^+^ macrophages and GR1^+^ neutrophils in lung tissue 7 days after S1 instillation. Quantification of MAC2^+^ and GR1^+^ cells disclosed a significant accumulation of macrophages (**Figure 2D**) and neutrophils (**Figure 2E**) in the lung of mice receiving S1 pre-incubated with saline compared to mice treated with vehicle. Pre-incubation of S1 with ASA effectively dampened the infiltration of inflammatory cells in the lungs of hACE2-KI mice (**Figures 2D, E**).

To rule out the possibility that ASA present in the pre-treated S1 preparation was responsible for the protective effects, an additional group of animals received intratracheal instillation of untreated S1 followed immediately by intratracheal delivery of 15 μl ASA 20 mg/L. Under this condition, S1 followed by ASA intratracheal administration still induced lung injury (**Figure 2A**), fibrosis (**Figures 2B, C**) and inflammatory cell infiltration (**Figures 2D, E**). This lack of protection confirms that ASA acts through direct structural modification of the S1 protein and excludes indirect anti-inflammatory effects of ASA in the context of the rapid, single-bolus exposure characteristic of our acute instillation model.

Taken together, these findings suggest that ASA has the potential to interfere with the interaction between the S1 protein and ACE2, thus attenuating S1-induced damage across ACE2-expressing tissues and limiting its widespread pathogenic effects.

### Treatment with ASA alters the glycosylation profile of S1

**Figure 3:**
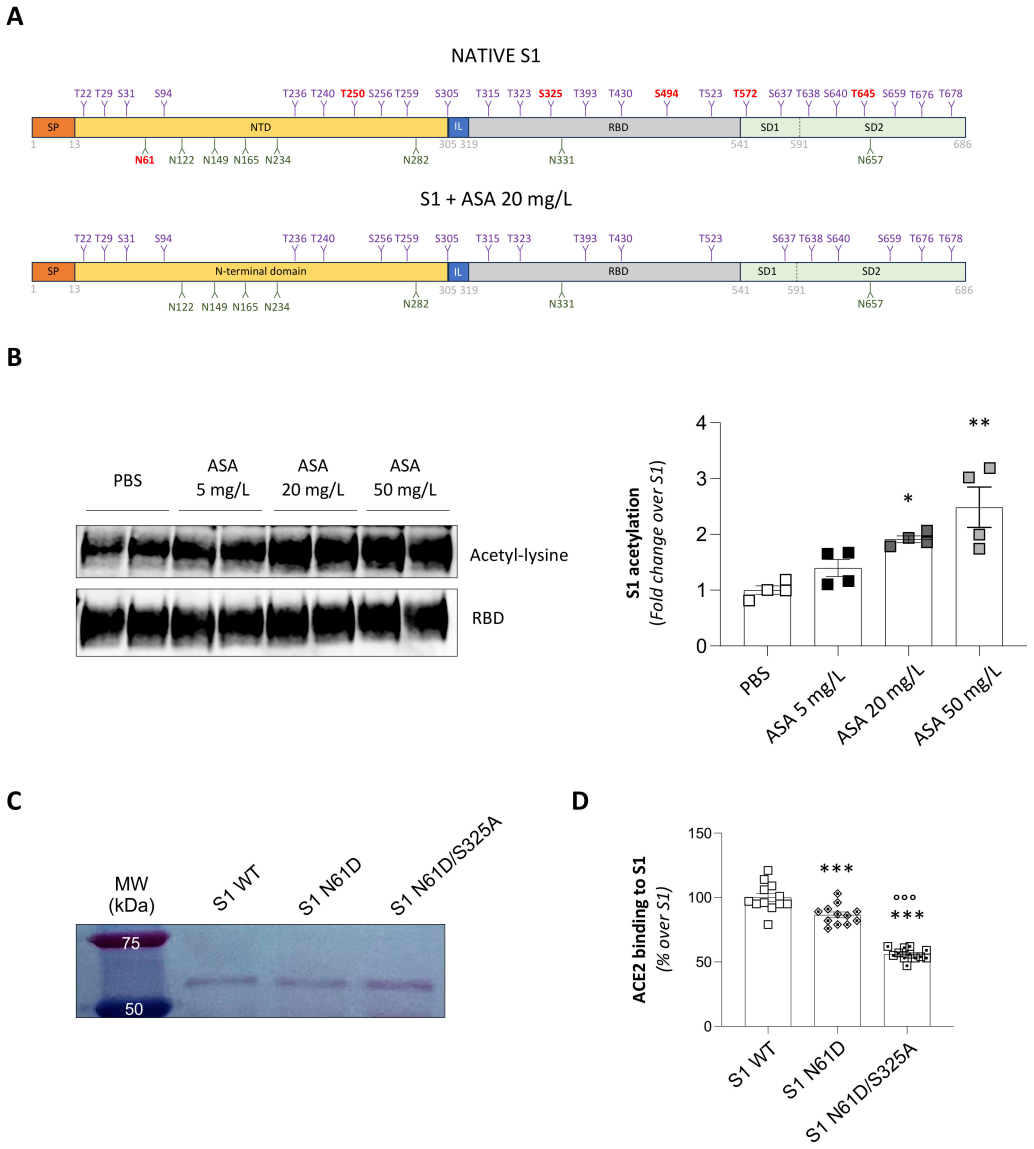
Structural mapping and functional relevance of glycosylation sites on SARS-CoV-2 S1 protein upon ASA treatment. **(A)** Schematic representation of the SARS-CoV-2 Spike S1 protein, comprising a signal peptide (SP, aa 1–13), the N-terminal domain (NTD; aa 14–305) a short interdomain linker (IL, aa 306–318), the receptor binding domain (RBD, aa 319–541), and subdomain 1 (SD1, aa 542–591) and subdomain 2 (SD2, aa 592–686). Glycoproteomic profiling revealed 8 N-linked and 25 O-linked glycosylation sites across different domains of S1. Treatment of the S1 with ASA 20 mg/L selectively removed N-glycosylation at residue N61 and O-glycosylation at residues T250, S325, S494, T572, and T645 (indicated in red). **(B)** Representative Western blot and quantification of S1 acetylation after overnight incubation in PBS or with increasing concentrations of ASA (ASA 5 mg/L, ASA 20 mg/L, and ASA 50 mg/L). Acetyl-lysine levels were detected using a rabbit anti-acetyl-lysine antibody, while a mouse anti-RBD antibody was used as loading control for normalization. Results are shown as mean ± SEM and were analyzed with Tukey’s multiple comparison test. *p-value<0.05, and **p-value<0.01 *vs* PBS. **(C)** Western blot analysis of wild-type S1 (S1 WT), mutant S1 N61D, and the double mutant S1 N61D/S325A, confirming successful expression and comparable molecular weight of the produced recombinant proteins. **(D)** Quantification of ACE2 binding to recombinant S1 proteins using the ELISA-based functional assay described in **Figure 1B**. Data are expressed as a percentage of ACE2 binding relative to S1 WT (n=12 *per* group). Results are shown as mean ± SEM and were analyzed with Tukey’s multiple comparison test. ***p-value<0.001 *vs* S1 WT; °°°p-value<0.001 *vs* S1 N61D.

To elucidate the mechanism by which ASA inhibits the binding of S1 to ACE2, we focused on S1 protein glycosylation. This hypothesis was grounded in the extensive glycosylation of the S1 protein, which plays a pivotal role in spike protein folding, immune evasion (62), and ACE2 engagement (63, 64), coupled with the previously reported antiglycation activity of ASA (60). To test this, we performed glycoproteomic profiling of S1 in the native state, as well as following treatment with 20 mg/L ASA to comprehensively define any potential ASA-induced glycosylation changes. As depicted in **Figure 3A**, the native S1 protein displayed 8 N-linked and 25 O-linked glycosylation sites (**Supplementary Tables 1** and **2**). In our experimental setting, ASA treatment selectively reduced N-linked glycosylation at N61 residue (**Figure 3A**), located in the N-terminal domain (NTD). In addition to this, ASA also limited O-linked glycosylation at multiple serine and threonine residues across distinct structural regions of the spike protein (**Figure 3A**). Specifically, we observed reduced O-linked glycosylation at T250 in the NTD, S325 and S494 within the RBD, and T572 and T645 in the subdomain 1 (SD1) and 2 (SD2).

Considering the well-known ability of ASA in inducing non-enzymatic acetylation of lysine residues (34), we next examined whether the observed changes in S1 glycosylation was accompanied by chemical modification of the S1 protein. To this end, we assessed S1 acetylation after exposure to increasing concentrations of ASA. As shown in **Figure 3B**, ASA induced a robust dose-dependent increase in S1 acetylation. This modification was evident at the lowest concentration tested (5 mg/L) and showed progressively strong enrichment at 20 mg/L and 50 mg/L (**Figure 3B**).

Altogether, these results indicate that ASA reshapes N- and O-linked glycosylation of the S1 protein, potentially through an acetylation-dependent process, thereby diminishing its capacity to bind ACE2.

### Site-directed mutagenesis confirms the critical role of glycosylation at N61 and S325 in mediating the interaction between S1 and ACE2

Among all the glycosylation sites modified by ASA, two specific residues, namely N61 and S325, have been previously described as potential modulators of S1-ACE2 interaction (63, 65). To assess the direct contribution of glycosylation at N61 and S325 in S1 binding to ACE2, we performed site-directed mutagenesis of the S1 protein through a PCR-based method. The pUNO1His-SARS2-S1 plasmid, containing the SARS-CoV-2 Spike S1 open reading frame, was used as a template. We introduced targeted amino acid substitutions of asparagine 61 with aspartic acid (N61D) to disrupt N-linked glycosylation, and substitution of serine 325 with alanine (S325A) to abrogate O-linked glycosylation. Besides the N61D plasmid, we generated the double mutant (N61D/S325A) by sequential mutagenesis using the N61D plasmid as a template for the second round. Sanger sequencing confirmed the correct mutation of the two residues (**Supplementary Figure 3**). WT and mutant proteins were purified from ExpiCHO cells transfected with the corresponding plasmids, and proper protein expression and purification was confirmed by Western blot analysis (**Figure 3C**). The resulting mutant proteins were used as coating antigens in the ELISA assay described in **Figure 1B** to assess ACE2 binding efficiency. In this experimental setup, the S1 protein featuring the N61D mutation showed a partial reduction in ACE2 binding efficiency when compared to its WT counterpart (**Figure 3D**). Notably, the double mutant harboring both N61D and S325A substitutions (N61D/S325A) exhibited an even more pronounced and statistically significant reduction in ACE2 binding efficiency than the single N61D mutation (**Figure 3D**).

Together, these findings provide direct evidence that simultaneous disruption of glycosylation at N61 and S325 is required to trigger a significant structural S1 rearrangement, hindering the recognition or accessibility to ACE2.

### Double S1 mutant has a limited pathogenic effect in transgenic mice with human ACE2

**Figure 4:**
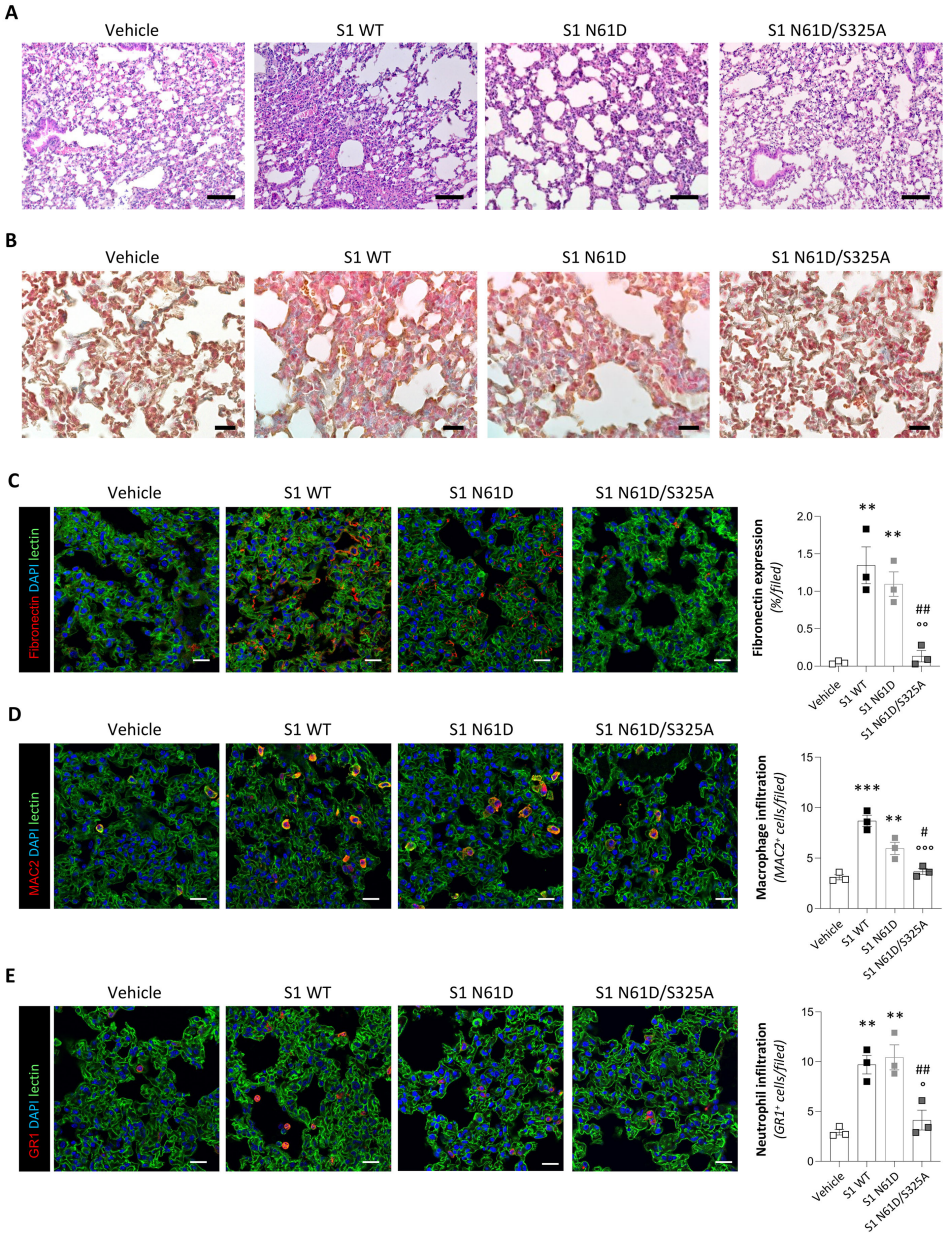
Glycosylation-deficient S1 double mutant displays reduced lung pathogenicity *in vivo*. **(A, B)** Representative images of lung sections stained with H&E **(A)** and Masson’s trichrome **(B)** from mice receiving intratracheal instillation of vehicle, 15 μg wild-type S1 (S1 WT), 15 μg single S1 mutant (S1 N61D) or 15 μg double S1 mutant (S1 N61D/S325A) at 7 days (n=3 *per* group). Scale bars: 100 µm for H&E and 20 µm for Masson’s trichrome staining. **(C–E)** Representative images and quantification of fibronectin (**C**, red), MAC2^+^ macrophages (**D**, red), and GR1^+^ neutrophils (**E**, red) in lung tissue sections of mice receiving intratracheal instillation of vehicle, S1 WT, S1 N61D or S1 N61D/S325A at 7 days (n=3 *per* group). Lung structures and nuclei were counterstained with WGA lectin (green) and DAPI (blue), respectively. Scale bar: 20 µm. Data are expressed as % of fibronectin fluorescence area *per* high power field at ×63 magnification (% area/field) and the average number of MAC2^+^ or GR1^+^ cells *per* high power field at ×63 magnification. For all panels, results are shown as mean ± SEM and were analyzed with Tukey’s multiple comparison test. **p-value<0.01, and ***p-value<0.001 *vs* Vehicle; °p-value<0.05, °°p-value<0.01, and °°°p-value<0.001 *vs* S1 WT; ^#^p-value<0.05, and ^##^p-value<0.01 *vs* S1 N61D.

To evaluate whether the reduced binding to ACE2 caused by the N61D/S325A double mutation affects the pathogenicity and organ damage induced by the S1 protein *in vivo*, we administered the mutated recombinant S1 intratracheally into hACE2-KI mice. In stark contrast to the S1 WT, hematoxylin and eosin staining of lung tissue 7 days after administration of S1 N61D/S325A double mutant revealed a marked attenuation of histopathological features (**Figure 4A**). Specifically, lungs from these mice exhibited well-preserved alveolar architecture, with minimal thickening of the alveolar walls and no evidence of diffuse parenchymal hypercellularity or vascular congestion (**Figure 4A**). Consistently, Masson’s Trichrome staining showed a near-complete absence of collagen deposition in lungs exposed to the mutated S1 protein, with only sparse fuchsin- and aniline blue-positive areas compared to the extensive remodeling induced by S1 WT (**Figure 4B**). This was paralleled by significantly reduced fibronectin expression in the lungs of mice receiving S1 N61D/S325A double mutant (**Figure 4C**). Furthermore, analysis of inflammatory cells revealed limited infiltration of MAC2^+^ macrophages (**Figure 4D**) and GR1^+^ neutrophils in the lungs of mice receiving S1 N61D/S325A double mutant compared to S1 WT (**Figure 4E**), with cell counts comparable to vehicle-treated mice. In line with the *in vitro* data, the single-mutant S1 N61D was less effective in reducing the pathogenicity *in vivo* than the double mutant. Indeed, S1 N61D induced lung injury (**Figure 4A**), fibrosis (**Figures 4B, C**), and inflammatory cell infiltration (**Figures 4D, E**) at levels comparable to S1 WT, underscoring that the protective phenotype is specific to double N61D/S325A substitution.

Altogether, these results demonstrate that the N61D/S325A double mutation, but not the single-mutant S1 N61D, mirrors the protective effects observed with ASA-treated S1, effectively preventing the organ damage and pathogenic responses induced by wild-type S1, further underscoring the specificity and functional relevance of the selected sites.

## Discussion

In the present study, we identify a novel antiviral activity of ASA that directly impairs the interaction between the S1 protein of SARS-CoV-2 and the human ACE2 receptor. Using a defined, cell-free system with recombinant S1 protein, we show that treatment with clinically relevant plasmatic concentrations of ASA (58) impairs S1–ACE2 binding independently of cellular metabolism or enzymatic processing. This experimental setup also allowed us to avoid the confounding influence of additional intracellular effects of ASA on host signaling pathways activated during SARS-CoV-2 infection, including NF-κB activation (47). These observations point to a distinct, non-canonical molecular mechanism of ASA in blocking S1 interaction with ACE2, separate from the well-established anti-inflammatory and antithrombotic effects of ASA.

This is of critical relevance considering that, beside facilitating viral entry, engagement of ACE2 by S1 triggers multiple detrimental signaling pathways known to participate in cellular responses, including inflammation, fibrosis and tissue damage (66), collectively contributing to COVID-19 severity. Therefore, the ability of aspirin to interfere with S1-ACE2 interaction and the consequent intracellular signaling may represent a critical mechanism to mitigate virus-induced tissue injury.

To assess whether the reduced S1-ACE2 binding induced by ASA effectively translates into meaningful tissue protection *in vivo*, we took advantage of mice transgenic for human ACE2 to maximize the binding of S1. Consistent with previous findings (67), intratracheal instillation of untreated S1 elicits marked histological lung damage, recapitulating key pathological features of COVID-19-related lung injury, including fibrosis and inflammation. In this setting, pre-incubation of recombinant S1 protein with ASA is sufficient to attenuate pulmonary injury, fibrosis, and inflammation in hACE2-KI mice, supporting a previously unreported role for ASA in modulating spike–receptor engagement. This is particularly relevant given that elevated levels of circulating SARS-CoV-2 antigens, especially the S1 subunit, are found in a significant proportion of patients during infection, correlating with cardiopulmonary symptoms (48, 49), and persist in some cases during the post-acute phase, contributing to the pathophysiology of long COVID (50, 51). Our findings may indicate that ASA inhibits circulating SARS-CoV-2 or viral antigens from binding to the host cellular receptor, thereby reducing tissue damage across ACE2-expressing organs. The broad tissue distribution of ACE2, which underlies the organ tropism and tissue-specific damage observed in severe SARS-CoV-2 infection (68), suggests that ASA may exert protective effects across multiple susceptible tissues by directly interfering with spike–ACE2 binding.

To uncover the molecular basis for these observed effects, we focused on protein glycosylation. This post-translational modification is particularly relevant for SARS-CoV-2. Indeed, the virus exhibits extensive glycosylation of the S protein (63, 64), which plays a crucial role in proper protein folding, interaction with the host receptor, and evasion of the immune response (62). On top of that, ASA has been shown to exert antiglycation properties (60), raising the intriguing possibility that this mechanism underlies its ability to disrupt the binding of the S1 protein to ACE2. To investigate this hypothesis, we conducted a comprehensive glycopeptidomic analysis comparing native S1 protein with ASA-treated S1. In our experimental setting, we observed 8 occupied N-linked sites in the native S1 protein, a lower number compared to previous reports. Indeed, Watanabe and colleagues identified 13 putative N-glycosylation sites within the S1 domain using the trimeric full-length Spike protein expressed in HEK293F cells (69). In contrast, Sanda et al. reported 11 N-glycosylation sites when analyzing recombinant full-length Spike expressed in HEK293 cells (70). These differences likely reflect the well-recognized variability introduced by differences in Spike protein constructs (e.g., pre-fusion stabilized trimers, full-length protein, recombinant S1 protein), expression systems (e.g., HEK293, CHO), and glycoproteomic workflows (70–74). The possibility that distinct spike constructs exhibit differential N-glycan occupancy is exemplified by findings from Shajahan et al., who used a recombinant S1 protein expressed in HEK293 cells and observed that several of the predicted N-glycosylation sites remained unoccupied in a significant proportion of peptides (75), a pattern consistent with our observations using a similar recombinant S1 protein. As for O-glycosylation, our glycopeptidomic analysis revealed a higher number of O-linked sites in the natively expressed recombinant S1 compared to previous studies. For instance, Shajahan and colleagues identified 9 O-glycopeptides in SARS-CoV-2 S1 protein produced by HEK293 cells (75). However, Tian et al. reported a total of 17 *O*-glycosites on the spike protein extracted from SARS-CoV-2 virions (76). In line with this latter finding, our identification of a higher number of O-linked sites highlights the enhanced sensitivity of our analytical workflow and corroborates the view that the O-glycosylation profile of recombinant S1 may be more intricate than previously appreciated.

Irrespective of these inherent variations in native spike protein glycosylation, our data crucially revealed loss of specific glycan structures on S1 following treatment with ASA. Among these, we found that ASA impacted N- and O-glycosylation at N61 and S325, which have been previously implicated as critical modulators of S1–ACE2 interaction (63, 65). Indeed, N61 is a conserved N-glycosylation site (N-X-S/T motif), consistently reported across multiple structural and glycoproteomic dataset (63, 73). Although distal from the RBD, the N-glycosylation at N61 is proximal to the furin-site and may increase the steric hindrance for cleavage, which seems to be beneficial to SARS-CoV-2 entry (63, 77). Additionally, S325 was recently unambiguously identified as an unexpected O-glycosylation site within the RBD (75). This residue has since been validated by multiple independent datasets (69, 73, 78–82), and proposed to influence spike–ACE2 binding affinity (75, 83, 84). Consistent with this, pharmacological inhibition of both N-linked and O-linked glycosylation significantly impairs viral entry (77), further supporting our findings that alterations in S1 glycosylation by ASA decrease its binding affinity for ACE2.

To directly test whether these changes in the glycosylation of these two critical residues have an impact on the binding activity of the spike protein, we performed site-directed mutagenesis experiments to substitute the asparagine (N) residue at position 61 with aspartic acid (N61D) and the serine (S) residue at position 325 with alanine (S325A), both of which prevent glycosylation at these sites. *In vitro*, the single mutation at residue N61 negatively modulated the binding activity of S1 to ACE2. Our data are in line with a previous study showing that single N61D mutation decreases S1 protein binding on the surface of cells (85). Adding another layer of complexity, our findings provide evidence that diminished O-glycan occupancy at S325 synergizes with the N61D mutation in reducing S1–ACE2 binding, thereby unveiling a previously unreported regulatory level in spike–host receptor interaction. The biological relevance of these two glycosylation sites on the functional properties of S1 was substantiated by *in vivo* experiments in hACE2-KI mice in which intratracheal administration of the N61D/S325A double mutant failed to induce significant alveolar damage, fibrotic remodeling, and immune cell recruitment. The convergence of results obtained with the mutated S1 and with ASA pre-treatment strengthens the hypothesis that interfering with S1 glycosylation at the ACE2 binding interface, either structurally or pharmacologically, is sufficient to mitigate the pathogenic activity of S1 in pulmonary tissue.

Altogether, these findings highlight a glycosylation-based mechanism through which ASA impairs its functional interaction with ACE2, ultimately exerting antiviral effects. Given that our system involved recombinant spike protein exposed directly to ASA, the observed glycosylation changes are strongly indicative of non-enzymatic chemical modifications. In this context, a plausible explanation involves the well-documented acetylating capacity of ASA (86), which may interfere with glycosylation through multiple mechanisms, including local conformational changes, or interactions with glycan chains themselves. In line with this hypothesis, in our experimental setting we found a detectable, dose-dependent increase in S1 acetylation upon ASA treatment. These findings support a structural mechanism by which ASA-induced acetylation may influence the local glycosylation environment. Importantly, both regions surrounding N61 and S325 contain multiple lysine residues that could potentially undergo ASA-dependent acetylation and contribute to these effects. Further studies, such as mass spectrometry, could elucidate the precise molecular interactions between ASA and spike protein, shedding more light on these novel findings.

From a clinical perspective, these insights complement the well-established anti-inflammatory and antithrombotic actions of ASA and offer a mechanistic rationale to guide the repurposing of ASA as an effective intervention to prevent the progression from mild to severe COVID-19. Although this study employed a recombinant S1 protein based on the original Wuhan-Hu-1 SARS-CoV-2 sequence, recent global sequencing efforts indicate that the N61 and S325 residues remain highly conserved across major circulating variants (64, 87, 88), including the more recent Omicron lineages. Crucially, recent site-specific glycoproteomic analyses have shown that both N- and O-glycosylation sites on the spike protein are largely conserved across major SARS-CoV-2 variants (89). This degree of conservation suggests that the modulatory effects of ASA on S1 glycosylation and its subsequent impact on ACE2 binding may therefore have clinical relevance beyond the ancestral strain. However, given the substantial amino acid diversification observed among SARS-CoV-2 variants, future studies should evaluate whether variant-specific residue substitutions, including the introduction or loss of lysine residues, may modulate the susceptibility of S1 to ASA-induced acetylation and, consequently, its inhibitory effect on ACE2 binding across different viral lineages. Finally, these findings may also have broader implications for other viral infections in which glycosylation plays a critical role in mediating virus–host interactions, such as influenza (90, 91).

In conclusion, these insights expand the current understanding of the pharmacological profile of ASA and lay the groundwork for potential repurposing strategies against emerging respiratory viruses with glycosylation-dependent entry mechanisms.

## Materials and methods

### Study approval

All procedures involving animals were performed in accordance with institutional guidelines in compliance with national (D.L.n.26, March 4, 2014), and international laws and policies (directive 2010/63/EU on the protection of animals used for scientific purposes). This study was approved by the Institutional Animal Care and Use Committees of Istituto di Ricerche Farmacologiche Mario Negri IRCCS and by the Italian Ministry of Health (approval number 371/2023-PR).

### Animal experiments

A total of 24 male hemizygous B6.129S2(Cg)-Ace2^tm1(ACE2)Dwnt/J^ (hACE2-KI) mice (strain #:035000; The Jackson Laboratory) were maintained in a specific pathogen-free facility at a constant temperature with a 12:12-h light/dark cycle and free access to a diet and water, as we previously described (92, 93). At 12 weeks of age, n=9 hACE2-KI mice were anesthetized with isoflurane (4–5% induction, 2–3% maintenance) and randomly allocated to receive a 15 μL intratracheal instillation of 15 μg SARS-CoV-2 spike protein S1 produced in HEK293 cells (Invivogen, his-sars2-s1) incubated overnight in saline (S1, n=3 mice) or with 20 mg/L ASA (ASA-treated S1, n=3 mice). One group of animals received a 15 μL instillation of untreated S1 followed by ASA administered immediately afterward through the same route, serving as a control to exclude any confounding effect of residual ASA from the incubation step (S1 + ASA, n = 3 mice). In an additional experiment, n=9 hACE2-KI mice were randomly allocated to receive a 15 μL intratracheal instillation of 15 μg of S1 wild-type (S1 WT, n=3 mice), single S1 double mutant (S1 N61D, n=3 mice) or double S1 double mutant (S1 N61D/S325A, n=3 mice). In both settings, hACE2-KI mice receiving an intratracheal instillation of 15 μL saline served as controls (Vehicle, n = 6 mice). No mortality was observed throughout the experimental period. Following 7 days, mice were euthanized by CO_2_ at a flow rate of 30% of the chamber volume *per* minute (Quietek™ 1, Next Advance, Inc.) and lungs processed for subsequent analyses. The researchers conducting the procedures on the animals and all subsequent analyses were blinded to the treatments. No inclusion or exclusion parameters were used. The work has been reported in line with the 2.0ARRIVE guidelines (94).

### Lung histology and fibrosis

Lung samples were fixed in formalin. Paraffin-embedded sections (4-μm thick) were stained with hematoxylin–eosin and assessed by light microscopy. Lung fibrosis was evaluated on paraffin-embedded sections stained with Masson’s trichrome to highlight collagen deposition.

### *In vivo* immunofluorescence analysis

Optimum Cutting Temperature (OCT)-frozen lung sections (4-μm thick) were fixed in paraformaldehyde (PFA) 4% incubated with 1% BSA to block nonspecific sites. The sections were then incubated with the rabbit anti-fibronectin (1:600; Abcam, ab2040), rat anti-GR1 antibody that recognizes the lymphocyte antigen 6 complex locus G6D (Ly6G; 1:50; Caltag, RM3000), rat anti-macrophage antigen 2 (MAC2; 1:200; Cederlane, CL8942AP) followed by the corresponding Cy3-conjugated secondary antibodies (Jackson ImmunoResearch Laboratories), as appropriate. Negative controls were obtained by omitting the primary antibody on adjacent sections. Lung structure was counterstained with FITC-wheat germ agglutinin (WGA) lectin (Vector Laboratories, FL-1021). Nuclei were counterstained with 4’,6-diamidino-2-phenylindole (DAPI, Sigma-Aldrich). Samples were examined under confocal inverted laser microscopy (Leica TCS SP8, Leica Microsystems). The expression of fibronectin in the lung was quantified with ImageJ (version 1.40g). Digitized images were binarized using a threshold, the values were expressed as the percentage of the area occupied by the staining *per* total area in an average of 10 high-power fields at ×63 magnification. For quantification of neutrophil and macrophage infiltration, GR1^+^ or MAC2^+^ cells were counted in an average of 10 high-power fields at ×63 magnification and expressed as the average number of cells *per* field.

### Cell culture and incubations

VERO C1008 (Vero 76, cloneE6, Vero E6; ATCC, CRL1586; RRID: CVCL_0574) were cultured in Eagle’s minimal essential medium (EMEM; ATCC, 302003) supplemented with 10% heat inactivated fetal bovine serum (FBS) and 1X penicillin/streptomycin (P/S).

For cell viability studies, VERO C1008 cells were seeded at 80.000 cells/cm^2^ in 24 well plate. At confluence, cells were exposed to control medium alone (EMEM supplemented with 2% FBS, 1X P/S) or with S1 at a concentration of 10 nM, 20 nM, and 50 nM in the presence of anti-ACE2 functional blocking antibody (α-ACE2, 2 μg/ml, Adipogen, AG-20A-0037PF) or the corresponding normal mouse IgG (IRR; Santa Cruz, sc-2025). After 24 hours, cells were detached and counted.

### Immunofluorescence analysis

For immunofluorescence analysis, VERO C1008 cells were seeded 80.000 cells/cm^2^ on coverslips. At confluence, cells were incubated for 24 hours with either control medium or 20 nM of the S1 protein that had been pre-incubated overnight with control medium or with acetylsalicylic acid (ASA; Sigma-Aldrich, A5376) diluted in at concentrations of 5, 20, or 50 mg/L. In selected experiments, 20 nM of the S1 protein was incubated with 20 mg/L paracetamol (PARA; Sigma-Aldrich, P0300000). After treatments, cells were fixed in 2% paraformaldehyde (PFA, Electron Microscopy Science) and 4% sucrose (Sigma-Aldrich) for 20 minutes. Non-specific binding sites were blocked with 2% FCS, 2% BSA, and 0.2% bovine gelatine in PBS 1X. Cells were incubated with a mouse anti-SARS-CoV-2 RBD (1:1,000; Abcam, ab277624), followed by incubation with a goat anti-mouse Cy3-conjugated (1:80; Jackson ImmunoResearch Laboratories). Negative controls were obtained by omitting primary antibodies. Nuclei were counterstained with DAPI (Sigma-Aldrich). After mounting with Dako (Agilent Technologies), cover slips were examined using confocal microscope.

The binding of S1 to VERO C1008 was quantified with ImageJ. Digitized images were binarized using a threshold, the values were expressed as the percentage of the area occupied by the staining *per* total area in an average of 10 high-power fields at ×63 magnification.

### Infectivity assay

For SARS-CoV-2 infection, VERO C1008 cells were seeded at 80.000 cells/cm^2^ in 48 well plate, as we previously described (95). To evaluate the infection potential, 200 µl of SARS-CoV-2 positive nasopharyngeal swab (UTM™, Copan Italia) was preincubated or not with 20 mg/L ASA for 1 hour and inoculated on confluent VERO C1008 cells in 300 µl of EMEM, 2% FBS, 1X P/S, and 5 μg/mL TPCK-treated trypsin (Sigma Aldrich). After 2-hour incubation at 37 °C in 5% CO_2_, infection medium was discarded and 500 µl of fresh EMEM, 2% FBS, 1X P/S, and 5 μg/mL TPCK-treated trypsin was added to each well. Cells were observed daily for evidence of cytopathic effects.

### ELISA assay

ELISA-based binding activity of ACE2 to S1 protein was adapted with minor modifications from previously published protocols (59). Briefly, 96-well plates (Bethyl Laboratories, E101) were coated with 200 ng S1 incubated or not with 10 mg/L, 20 mg/L, or 50 mg/L ASA in PBS 1X overnight. In additional experiments, 200 ng of S1 wild type (S1 WT), single S1 mutant (N61D), or double S1 mutant (N61D/S325A) were used as coating antigens. The following day, wells were washed and incubated with blocking buffer (50 mM Tris buffered saline, pH 8.0, 1% BSA; Bethyl Laboratories, E101) for 30 minutes at room temperature. Then, wells were incubated with 0.1 μg/mL ACE2 with C-term human Fc tag (Invivogen, fc-hace2) for 1 hour at room temperature in PBS 1X. A rabbit anti-spike antibody (Abcam, ab275759) was used as positive control. After washing, wells were incubated with a goat anti-human Fc HRP affinity pure (Invitrogen, A18817). The chromogenic reaction was quantified following the addition of TMB substrate and stop solution (Bethyl Laboratories, E101). The absorbance of the samples was measured on the multimode microplate reader TECAN Infinite M200^®^ PRO at 450 nm with a reference wavelength of 650 nm.

### Glycopeptidomics and glycosylation site mapping of SARS-CoV-2 S1 protein

Glycopeptidomics analysis was performed by Creative Proteomics (Shirley, NY, USA) on recombinant SARS-CoV-2 S1 proteins (untreated and ASA-treated), expressed in HEK293 cells. Approximately 100 µg of each protein sample was processed using the following workflow. Proteins were first buffer-exchanged using Microcon YM-10 centrifugal filter units (Millipore), followed by reduction with 10 mM dithiothreitol (DTT) at 56 °C for 1 hour and alkylation with 20 mM iodoacetamide (IAA) at room temperature in the dark for 1 hour. After removal of reagents by centrifugation and washing with 50 mM ammonium bicarbonate, the proteins were digested overnight at 37 °C with sequencing-grade trypsin (Promega) at an enzyme-to-substrate ratio of 1:50. The resulting peptides were recovered by centrifugation and lyophilized to near dryness. Prior to LC-MS/MS analysis, peptides were reconstituted in 20 µL of 0.1% formic acid. Peptides were analyzed using an Ultimate 3000 nano UHPLC system coupled to a Q Exactive HF mass spectrometer (Thermo Fisher Scientific) with an ESI nanospray source. Peptides were loaded onto a trapping column (PepMap C18, 100 Å, 100 µm × 2 cm, 5 µm) and separated on an analytical column (PepMap C18, 100 Å, 75 µm × 50 cm, 2 µm) using a linear gradient of 0.1% formic acid in 80% acetonitrile at a flow rate of 250 nL/min. The gradient progressed as follows: 2–8% B in 3 min, 8–20% B in 50 min, 20–40% B in 43 min, and 40–90% B in 4 min. The MS full scan was acquired over 300–1650 m/z at a resolution of 60,000 (at 200 m/z), with an AGC target of 3e6. MS/MS spectra were acquired in Top20 mode with a resolution of 15,000, AGC target of 1e5, maximum injection time of 19 ms, and normalized collision energy of 28%. Dynamic exclusion was set to 30 s. Singly charged and >6-charged ions were excluded. Raw MS data were analyzed using Byonic software, with the protein sequence provided by the client as the reference. A glycopeptide score threshold of ≥300 was applied to ensure high-confidence identifications. N- and O-linked glycosylation sites were mapped across the S1 protein.

### Site-directed mutagenesis

Site-directed mutagenesis was performed to introduce specific amino acid substitutions in the S1 protein of the SARS-CoV-2 Spike protein, encoded in the pUNO1His-SARS2-S1 plasmid (InvivoGen, p1his-cov2-s1), targeting predicted glycosylation sites. Mutations were introduced using the Phusion™ Site-Directed Mutagenesis kit (Thermo Fisher Scientific, F541) following the manufacturer’s instructions. For each mutation, specific mutagenic primers were designed with the desired nucleotide substitution centrally located. Briefly, the asparagine at position 61 was replaced with aspartic acid (N61D) to disrupt N-linked glycosylation, and serine at position 325 with alanine (S325A) to abrogate O-linked glycosylation. The optimal annealing temperature for each primer pair was calculated using the Thermo Fisher Scientific Tm Calculator tool (available at: thermofisher.com/tmcalculator). For the generation of the double mutant (S1 N61D/S325A), the plasmid containing the N61D mutation was used as the template for a second round of mutagenesis using the primer pair specifically designed for the S325A mutation. The final reaction products were transformed into One Shot™ TOP10 Chemically Competent E. coli cells (Thermo Fisher, C4040-10) by heat shock at 42 °C for 30 seconds. Transformed cells were plated on LB low salt agar containing 100 µg/mL of Blasticidin (InvivoGen, ant-bl-1) as the selective antibiotic, and insertion of the N61D and N61D/S325A mutations, together with the absence of any non-specific mutations, was confirmed by Sanger sequencing of the plasmids (purified through NucleoSpin Plasmid, Mini kit for plasmid DNA, Macherey-Nagel) using the BigDye™ Terminator v3.1 Cycle Sequencing Kit (Thermo Fisher Scientific), according to the manufacturer’s protocol. Sequencing analyses were carried out on the 48-capillary 3730 DNA Analyzer (Life Technologies). The results were aligned using SnapGene software. Large scale plasmid purification from the selected colonies was performed using EndoFree Plasmid Maxi kit (Qiagen, 12362), according to the manufacturer instructions. Sequences of primers used for mutagenesis and sequencing are reported in **Supplementary Table 3**.

### Expression and purification of S1 proteins in ExpiCHO cells

Confirmed wild-type and site-directed mutant plasmids of the SARS-CoV-2 spike S1 protein were transfected into ExpiCHO™ cells using the ExpiCHO™ expression system (Thermo Fisher Scientific, A29133) following the manufacturer’s instructions for optimized max-yield protein expression protocol. ExpiCHO-S™ cells were cultured in ExpiCHO Expression Medium in a humidified incubator at 37 °C, 8% CO_2_ in Erlenmeyer shaker flasks on an orbital shaker platform at a speed of 125 rpm. Cells were passaged every 2–3 days and kept in log-phase growth, ensuring viability >95% prior to transfection. For transfection, cultures were seeded at density of 6×10^6^ cells/mL in fresh pre-warmed medium and each plasmid DNA (1 µg/mL) was diluted in OptiPRO™ SFM and mixed with ExpiFectamine™ CHO reagent for 5 minutes at room temperature, according to the manufacturer’s instructions. After incubation to allow complex formation, the transfection mixture was added directly to the cultures. ExpiCHO™ Enhancer and Feed supplements were added at 24-hours post-transfection and cells were shifted to 32 °C and 5% CO_2_ with shaking. A second volume of ExpiCHO™ Feed was added on day 5 post-transfection to boost protein expression. Cultures were maintained for 12–14 days, and supernatants were harvested by centrifugation at 4000 × g for 30 minutes at 4 °C. Recombinant S1 proteins, both wild-type and mutated forms, were purified from the clarified supernatants by immobilized metal affinity chromatography (IMAC) using HisPur™ Ni-NTA Spin Purification kit (Thermo Scientific, 88229), equilibrated in 200 mM sodium phosphate, 3 M sodium chloride, pH 7.4, and eluted with 250 mM imidazole. Eluted proteins were concentrated using Centrifugal Filters NeoSpin 10 KD (CliniSciences, NB-57-0007), protein concentration was determined using the BCA Protein Assay Kit (Thermo Scientific) and diluted in saline to a final concentration of 1 mg/mL. Purity and integrity were assessed by Western blot. Specifically, 1 µg of each S1 WT and S1 mutant proteins was resolved on a 4–15% Mini-PROTEAN^®^ TGX™ Precast Protein Gels (Bio-Rad Laboratories, 4561084). Proteins were then transferred to 0.2 µm nitrocellulose membranes (Bio-Rad Laboratories, 1704159) and verified by Ponceau S staining.

### Western blot analysis of S1 acetylation

For the analysis of S1 acetylation, 1 µg of untreated or ASA-treated S1 was resolved on a 4–15% Mini-PROTEAN^®^ TGX™ Precast Protein Gels (Bio-Rad Laboratories) and transferred to 0.2 µm nitrocellulose membranes (Bio-Rad Laboratories). After blocking with 5% bovine serum albumin (BSA) in Tris-buffered saline supplemented with 0.1% Tween-20, membranes were incubated overnight at 4 °C with rabbit anti-acetylated lysine (1:1,000; Cell Signaling, #9441) and mouse anti-SARS-CoV-2 RBD (1:1,000; Abcam, ab277624). The signals were visualized on Odyssey^®^FC Imaging System (LiCor) by infrared (IR) fluorescence using a donkey anti-rabbit IRDye 680LT (1:1,000; LiCor, 926-68023) or a goat anti-mouse IRDye 800CW (1:1,000; LiCor, 926-32210) secondary antibody, as appropriate. Bands were quantified by densitometry using the Image Studio Lite 5.0 (LiCor) software.

### Sample size and statistical analysis

The sample size of the *in vivo* studies was estimated based on our preliminary data showing a marked increase in neutrophil infiltration in S1-treated animals (mean ± SD: 9.7 ± 3.0) compared to controls (1.6 ± 0.2). In this setting, we estimated an effect size (Cohen’s d) of ~3.8. Using standard power calculations (α=0.05, power=0.8), a minimum of 3 animals *per* group is sufficient to detect statistically significant differences.

All data are presented as the mean ± standard error of the mean (SEM). Data analysis was performed using Prism Software (GraphPad Software Inc). Comparisons were made using unpaired t-test or ANOVA with Tukey *post hoc* test, as appropriate. Statistical significance was defined as p-value<0.05. The sample size for each analysis is indicated in the corresponding figure legend. The datasets presented in this study can be found in the online repository Zenodo (10.5281/zenodo.17659820). 

## Data Availability

The original contributions presented in the study are included in the article/**Supplementary Material**. Further inquiries can be directed to the corresponding author.
