# Supplementary material for: Acetylsalicylic acid disrupts SARS-CoV-2 spike protein glycosylation and selectively impairs binding to ACE2
Source: Front Immunol. 2026 Jan 7;16:1706997. doi: 10.3389/fimmu.2025.1706997 (PMC12819676; doi:10.3389/fimmu.2025.1706997)
Supplement: Supplementary file 1 [file DataSheet1.pdf]

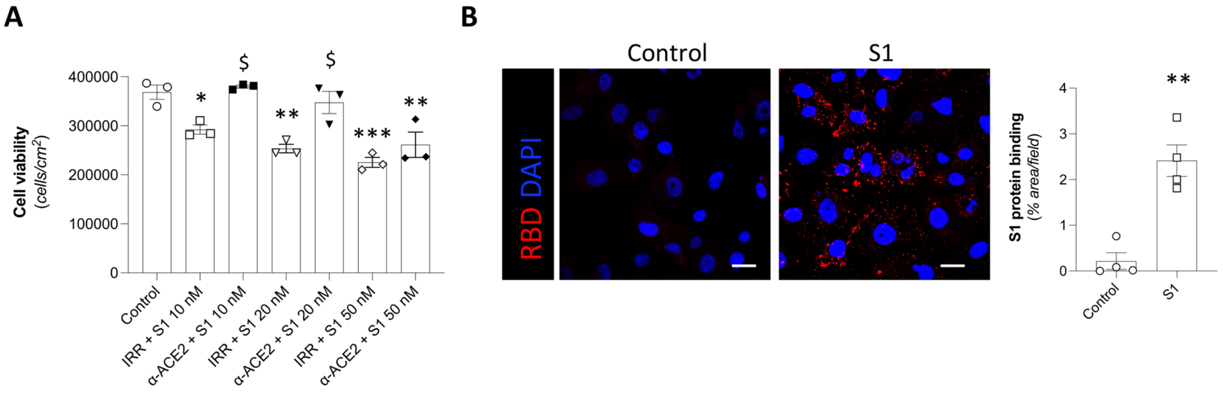

Supplementary Figure 1. Cytotoxic effect and S1 binding on Vero cells. (**A**) Quantification of cell viability in Vero cells exposed for 24 h to medium alone (Control) or S1 at the concentration of 10, 20, or 50 nM in the presence of an irrelevant isotype antibody (IRR; 2 µg/ml) or an ACE functional blocking antibody (α-ACE2; 2 µg/ml). Data are expressed as cell number *per* cm^2 (n=3 *per* group). (**B**) Representative images and quantification of the binding of S1 protein (RBD, red) on Vero cells following 24 h treatment with medium alone (Control) or S1 20 nM (S1). Nuclei were counterstained with DAPI (blue). Data are expressed as % of positive area *per* high power field at ×63 magnification (% area/field, n=4 *per* group). Scale bar 20 µm. Results are shown as mean ± SEM and were analysed with unpaired t-test or Tukey's multiple comparison test, as appropriate. *p-value<0.05, **p-value<0.01, and ***p-value<0.001 *vs* Control; $^{\$}$p-value<0.05 *vs* respective IRR + S1.

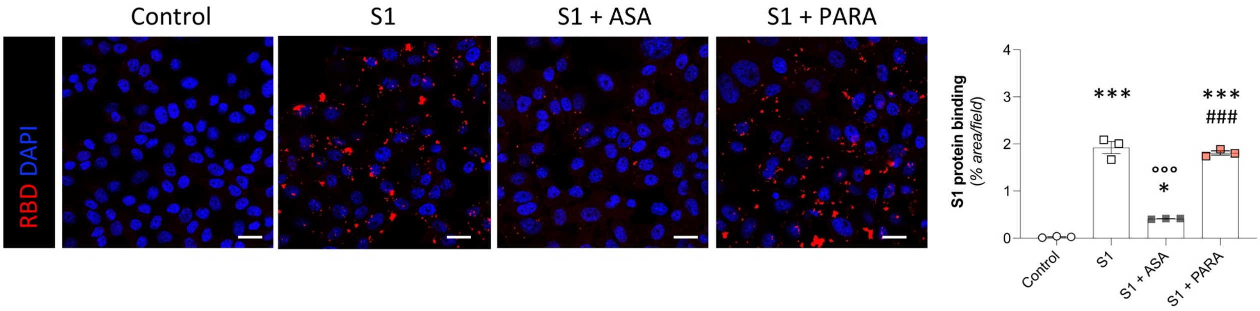

Supplementary Figure 2. Paracetamol does not inhibit S1 binding on Vero cells. Representative images and quantification of the binding of S1 protein (RBD, red) on Vero cells following 24 h treatment with 20 nM S1 incubated overnight with control medium (S1), ASA 20 mg/L (S1 + ASA), or 20 mg/L paracetamol (S1 + PARA). Cell treated with medium alone served as Control. Nuclei were counterstained with DAPI (blue). Data are expressed as % of positive area *per* high power field at ×63 magnification (% area/field, n=3 *per* group). Scale bar 20 µm. Results are shown as mean ± SEM and were analysed with Tukey's multiple comparison test, as appropriate. *p-value<0.05, and ***p-value<0.001 *vs* Control; °°°p-value<0.001 *vs* S1; ###p-value<0.001 *vs* S1+ASA.

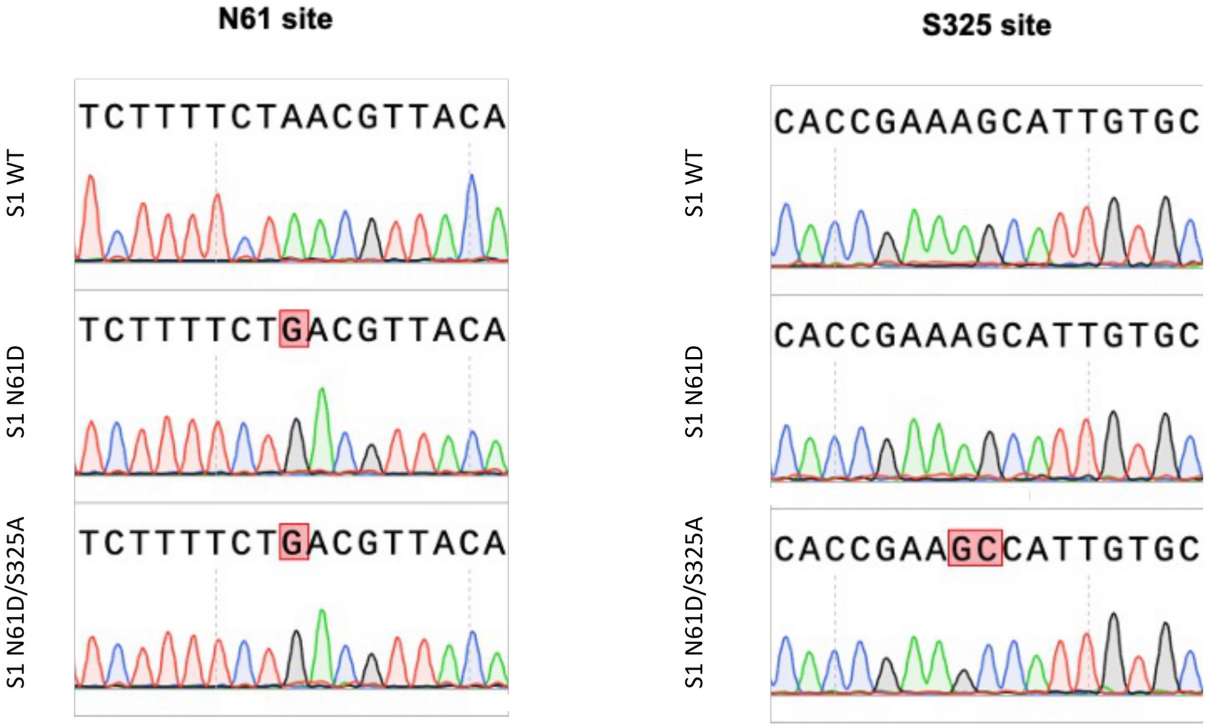

Supplementary Figure 3. Sanger sequencing confirmed of S1 mutants. Representative SnapGene alignments showing the nucleotide substitutions in S1 single mutant (S1 N61D) and S1 double mutant (S1 N61D/S325A) within the selected target sites: N61 (left panel) and S325 (right panel). Edited sequences are aligned against the wild-type S1 sequence (S1 WT) as reference.
