# Supplementary material for: Acetylsalicylic acid disrupts SARS-CoV-2 spike protein glycosylation and selectively impairs binding to ACE2
Source: Front Immunol. 2026 Jan 7;16:1706997. doi: 10.3389/fimmu.2025.1706997 (PMC12819676; doi:10.3389/fimmu.2025.1706997)
Supplement: Supplementary file 4 [file Table3.docx]

**Supplementary Table 3.** Primers for Site-directed Mutagenesis.

| **Primers for site directed mutagenesis** | | |
| --- | --- | --- |
| **YP_009724390** | **Nucleotide substitution in pUNO1His-SARS2-S1 plasmid** | **Primer sequence** |
| N61>D | AAC>GAC: c.193A>193G | Fwd: CCTTTCTTTTCT**G**ACGTTACATGGT  Rev: CAGAAAGAGGTCCTGAGTAGAGTG |
| S325>A | AGC>GCC: c.985AG>GC | Fwd: ACCCACCGAA**GC**CATTGTGCGG  Rev: TGGACCCGGAAATTACTGGTTTGGT |
| **Sequencing primers covering the SARS-CoV-2 Spike S1 ORF** | | |
| **Locus** | | **Primer sequence** |
| N61 containing locus | | Fwd: CTCCCTTGGAGCCTACCTAGA  Rev: TCACGTACTCGAAGGTGCAG |
| S325 containing locus | | Fwd: TGCACCTTCGAGTACGTGAG  Rev: GTCTCACTTCATCGCCTCGT |
| Primers covering the rest of the ORF to prove the lack of non-specific mutation insertions | | Fwd: CGGATGCTGTTGATTGTGCC  Rev: GGTAACCCACCCCGTTTGTA |
